# Adolescents and youths’ opinions about the factors associated with cannabis use: a qualitative study based on the I-Change model

**DOI:** 10.1186/s12912-023-01283-z

**Published:** 2023-04-12

**Authors:** María González-Cano-Caballero, María-Carmen Torrejón-Guirado, María Dolores Cano-Caballero, Isotta Mac Fadden, María-Del-Carmen Barrera-Villalba, Marta Lima-Serrano

**Affiliations:** 1 Department of Nursing, School of Nursing, Physiotherapy and Podiatry, University of Seville, Seville, 41009 Spain; 2 Department of Health Promotion, School for Public Health and Primary Care CAPHRI, Maastricht University, Maastricht, The Netherlands; 3 Deputy Directorate of Care. Virgen de las Nieves University Hospital, Granada, 18014 Spain; 4 Department of Nursing, Faculty of Health Sciences, University of Granada, Av. de la Ilustración, 60, 18071 Granada, Spain; 5 Pablo de Olavide University, Seville, 41013 Spain

**Keywords:** I-Change model, Nursing, Cannabis, Qualitative research

## Abstract

**Background:**

To learn about the experiences and opinions of adolescent non-consumers and regular cannabis users about cannabis use and the factors that determine its use, using the I-Change explanatory model as a basis.

**Methods:**

Qualitative methodology with a content analysis was used. Focus groups were conducted with adolescents who were non-regular cannabis users (those who had not tried cannabis or had only experimented with it before) and semi-structured interviews were conducted with adolescent and young adult in recovery who were in a detoxification program. A deductive analysis of the audio-recorded and transcribed interviews was performed, using the domains of the I-Change Model as a reference.

**Results:**

Personal problems, social problems or family problems can lead to cannabis use. There was a lack of knowledge and low risk perception about consumption of this drug. There are other factors that influence consumption, the perception of advantages, such as the feeling of freedom and the influence of the peer group. The consumption of this substance in girls is changing, becoming more and more equal to that of boys. The family has an important role to play in preventing drug use.

**Conclusion:**

Knowledge of these factors is of vital importance as a prior step to the development of efficient intervention measures adjusted to the needs identified and the characteristics of the population.

## Background

It is estimated that 200 million people consumed cannabis in 2019, thus making it the most widely used illegal drug worldwide. The highest levels of consumption occur in people aged between 18 and 25, but even in people who are 15 and 16 years old, the annual consumption of this drug is 4.7%, higher than in the general population, which is 3.9% [1]. In Spain, you can have up to 100 grammes of cannabis for self-consumption in private spaces, but it can never be consumed in public spaces. The sale and trafficking of this drug is prohibited [2]. According to the Survey on Alcohol and Drugs in Spain (EDADES, for its Spanish acronym), 37.5% of the population aged 15–65 report having consumed cannabis at least once in their lives, with a consumption percentage of 10.5% in the last year, which rises to 22.1% in the 15–24 age group [3]. If we look at the 2021 Survey on Drug Use in Secondary Education in Spain (ESTUDES, for its Spanish acronym) report, 28.6% of students between 14 and 18 years old have consumed cannabis at least once in their lives, and 22.2% in the last year and, being more frequent in boys in both cases. As for the consumption profile, the average age of first use for both genders was around 15 years old, being more frequent in boys. This survey found that 17.8% of those who have consumed cannabis in the last year are possible problem drug users (obtained a score of 4 or more on the Cannabis Abuse Screening Test, which qualifies them as possible problem users). The average amount of consumption reported for students who have consumed cannabis in the last 30 days is 3.3 joints/day. In the case of those considered to be problem users, the average was 5.1 joints/day [4]. This situation has direct consequences on adolescence, as the developing brain is more sensitive to the effect of drugs than that of adults [5]. Factors such as recent use, frequency and age of onset of cannabis use have been associated with future cognitive and emotional consequences [6]. Specifically, its use by individuals aged 15–16 is a risk factor for the onset of psychosis or schizophrenia, as well as for the possibility of worsening both pathologies [7, 8]. In addition, cannabis use has consequences such as addiction, vulnerability to other drugs or the development of respiratory or cardiovascular diseases [9].

**Fig. 1:**
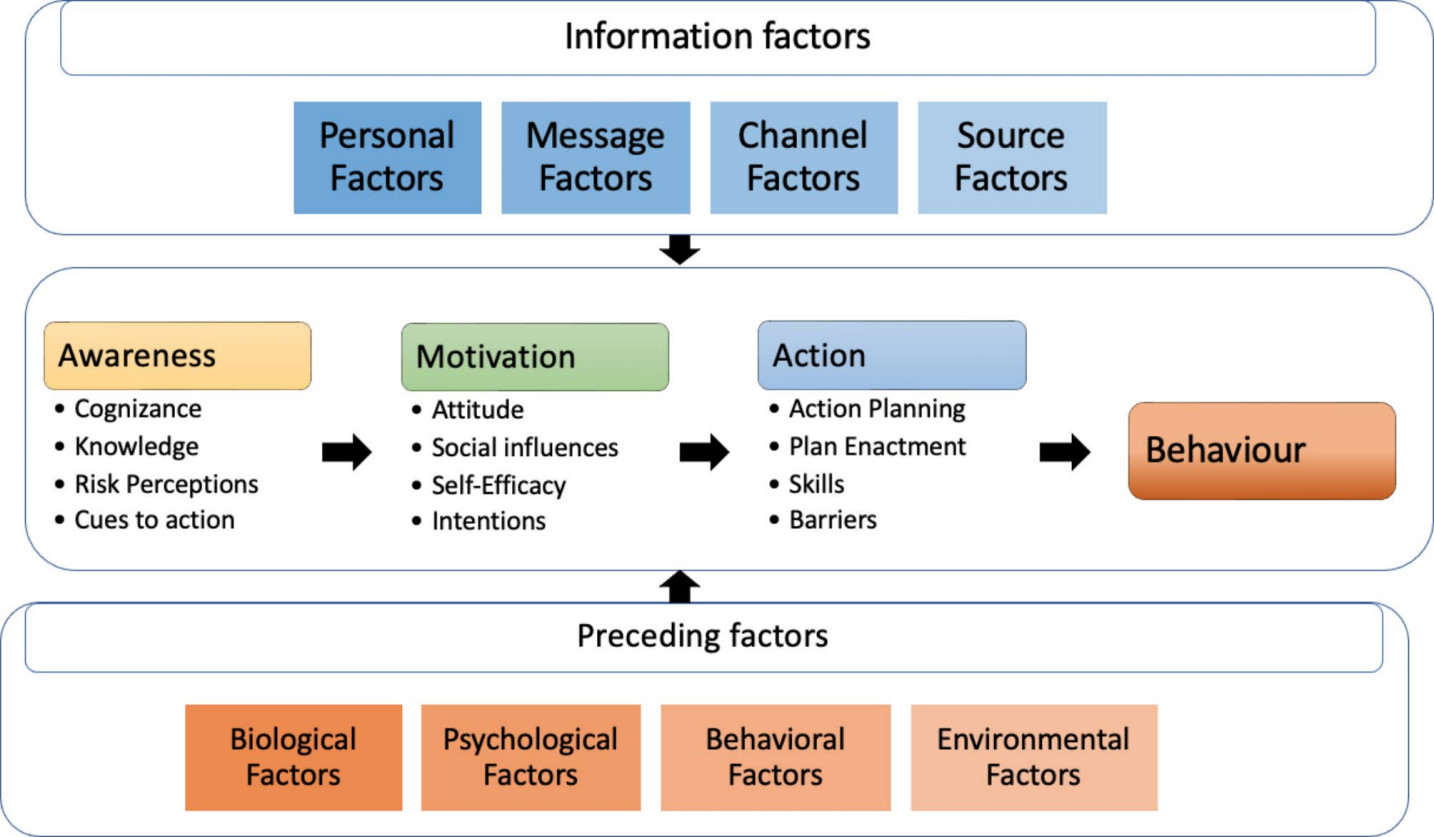
The I-Change Model 2017

In order to establish cannabis use prevention policies, it is necessary to delve deeper into this phenomenon and its deciding factors. To this end, this study takes the I-Change model as reference (Fig. 1), which integrates several previous theories to affirm that behavioral change moves through stages or phases where people go from being unaware of their behavior to deciding to change it [10]. This model distinguishes between informational factors (the quality of messages, channels and sources used), related to the communication process aimed at behavioral change, and individual factors classified as predisposing and motivational, the latter being the closest to behavioral change [11]. Predisposing factors are classified into aspects related to the individual (biological or psychological) or the environment (contextual) that determine motivation and, therefore, behavior [12, 13]. It is more difficult to influence these factors when developing prevention programs, but they must be taken into account during their design [11].

Motivation is divided into three phases: pre-motivational, motivational and post-motivational. The pre-motivational phase refers to awareness, and includes factors such as behavioral cognizance (being aware of one’s own behavior), knowledge (about the behavior/what information leads to taking action?), risk perception (perception of the possibility of a health threat and its severity) and cues to action (internal or external signs that initiate an action related to the behavior) [10].

The motivational phase includes attitude (the pros and cons of a given behavior), social influence [norms (norms or opinions of people close to the behavior), modeling (the influence of others who perform that behavior) and social pressure (the support that the individual finds in others to behave in a given manner)], self-efficacy (capacity that the person believes he/she has to carry out/avoid a given behavior), and intention [14].

The post-motivational or action phase is the phase closest to the behavior, and includes the realization of action-directed plans, the implementation of these plans (plan enactment), the person’s skills or barriers to action [12, 13].

[Figure 1. The I-Change Model 2017 [15].]

Although this model has not yet been used to analyze cannabis use in adolescents and young adults, it has served to explain other risk behaviors at these stages of life, such as smoking or drinking alcohol, as well as in the design of prevention programs for these behaviors [16–20], influencing the reduction of tobacco or alcohol consumption among adolescents [16, 17]. It is therefore considered useful for gaining in-depth knowledge of the factors involved in the behavior in order to propose effective intervention [19, 20]. On the other hand, the characteristics of qualitative research make it useful for understanding, from the perspective of those involved, the reasons linked to cannabis use [21]. Despite this, to date, there is little research that uses qualitative methodology to analyze the reasons that lead to cannabis use in adolescents and young adults. These studies highlight the influence of stressful events on cannabis use [22, 23] or the social influence of the peer group (friends and peers) on the onset and quit of drug use [22, 24, 25]. In addition, no studies have been found that make an in-depth exploration of all the factors involved in behavioral changes in people who used cannabis and that compare these same factors with those of non-consumers.

For all the above reasons, the aim of this research was to learn about the experiences and opinions of non-consumer adolescents and regular cannabis consumers on cannabis consumption and the factors that determine such consumption, using the I-Change explanatory model as a basis.

## Methods

### Design

The study was carried out using a qualitative content analysis, through Focus Groups and in-depth interviews, both conducted in Andalusia between 2017 and 2019. The recommendations of the COREQ guide were followed [26].

### Population and sample

Four Focus Groups were conducted with students aged between 14 and 17 (n = 20) who were enrolled in the 3rd and 4th years of compulsory secondary education (ESO), and who were also non-regular cannabis users (NRU) (those who had not tried cannabis or had only experimented with it before). In addition, semi-structured interviews were carried out with eight adolescents and young adult (AYA) aged between 16 and 20 who were also in recovery and who were enrolled in a detoxification program to quit cannabis use. Due to the characteristics of the detoxification center, we were only allowed to conduct individual interviews not focus groups.

The recruitment of the sample was based on a purpose sampling strategy. Table 1 shows the characteristics of the participants in the Focus Groups, and Table 2 shows the characteristics of the interviewees.

**Table 1:** Characteristics of the participants in the FG

		G1	G2	G3	G4
Sex	Male	3	4		
	Female	1		4	8
School Year		3rd ESO	4th ESO	4th ESO	4th ESO
Cannabis			1*		1*
Tobacco (No. of individuals)		1*	1*	1*	2 **
Alcohol (No. of individuals)		2**	2**	3**	4**

*tried the substances

**regular use

**Table 2:** Characteristics of the interviewees

	I1	I2	I3	I4	I5	I6	I7	I8
Sex	Male	Male	Male	Male	Female	Male	Male	Male
Age	18	16	22	17	17	20	16	19
Age at 1st use	15	15	18	15	13	15	13	15
Joints per day*	2 or 3	8	4 or 5	4 or 5	10 or 11	1 or 2	8 or 9	2
Tobacco consumption**	14 years old	13 years old	18 years old	12 years old	12 years old	16 years old	13 years old	14 years old
Alcohol consumption***	14 years old (weekends)	14 years old (rarely)	15–16 years old (daily)	12 years old (rarely)	12 years old (weekends)	14 years old (a beer/week)	14 years old (weekends)	14–15 years old (weekends)
Academic level	1st - high school (currently attending)	Primary (quit at 3rd ESO)	High-school	Secondary	Primary	Secondary	4th ESO (Currently attending)	Primary
Individuals he/she lives with	Mother and 3 siblings	Father, father’s partner and sibling	Father, mother and sister	Father, mother and sister	Father, mother and sister	Father, mother and 3 siblings	Mother	Mother, grandmother and 2 siblings
Occupation	Student	Construction worker	Student	Student	Cleaner and student	Waiter	Student	Farmer

* Joints per day when they were regular users before entering the program

**All of them are regular tobacco users. The age at onset is indicated

*** Alcohol consumption habits are prior to the start of the program, since while they are in the program it is forbidden for them to consume alcohol. The age at onset and frequency of consumption are indicated

### Instruments and data collection procedures

In both cases, a semi-structured script was used, based on the predisposing, awareness and motivational factors of the I-Change Model [10]. Although it is not part of the model, family factors have been included in this investigation because, being adolescents, families can influence the behavior of minors and, in this case, whether or not they consume cannabis. Open-ended questions were used, this allowed participants to express themselves freely, and in the case of the Focus Groups, ensured that the discussion would naturally flow. The questions in this script were modified to adapt them to each context. Table 3 shows the categories and questions used in each of the collection strategies. Informational factors were not explored in this study, as they are more concerned to educational aspects.

**Table 3:** Categories and questions used

Categories	NRU Questions	RU Questions
Cannabis use and consumption characteristics	Have you used cannabis? In what circumstances did such use take place?	When was the first time you used cannabis? Age. Situation. How it affected you. Consequences. Emotions.
Predisposing Factors	What aspects make a person start using cannabis? Do you think that the way girls use cannabis differs from that of boys? To what extent?	When you started using, what was your life like? What aspects make a person start using cannabis? Do you think that the way girls use cannabis is different from that of boys? To what extent?
Cognitive factors	What do you know about cannabis?	What did you know about cannabis before you started using? How has this knowledge changed from before you started using to now?
	To what extent do you consider the use of cannabis to be dangerous? What dangers and consequences do you perceive from the use of this substance? How likely would you be to suffer such consequence if you were to use cannabis?	To what extent did you consider cannabis use dangerous? How has this perception changed from before you started using it until now? What would you tell a friend, brother or sister if they asked you about this?
Motivational Factors	Attitude What advantages or disadvantages do you see in the use of cannabis? How could your opinion about the advantages change?	Advantages and/or disadvantages of cannabis use. How has this perception changed since before you started using until now?
	Social influence: model With whom do you usually use cannabis? Do you know people around you who use it?	What were your friendships like? Who did you hang out with? Did you change your friendships because of cannabis use? What was your opinion about people who used cannabis? How did you meet them? Did you know people around you who used cannabis? Who did you use cannabis with? How has this situation changed today? What are your friends and inner circle like?
	Social influence: social norm Who do you think approves cannabis use and who disapproves? Do the people around you think you should or should not use cannabis?	Were there any people around you who approved cannabis use? And did any people around you disapprove it? Were there any people whose opinion affected you? Why? Whose opinion affects you the most now?
	Social influence: social pressure Have you ever felt pressured to use cannabis? In what situation and with whom? If not, what situations do you think might put you under pressure to use? How could you act to avoid that situation or pressure from those people?	Have you ever felt pressured to use cannabis? If so, in what situation and by whom? If not, what situations do you think may put you under pressure to consume? Do you feel pressured by someone? How do you think you could act to avoid that situation or to avoid pressure from those people?
	Self-efficacy: In what situations do you think it is difficult to say no to cannabis use? What degree of difficulty do you face in each place (a party, a bar, a friend’s house, in the street, etc.)?	Situations that made it difficult to say no to cannabis use. Which do you consider to be risky for use?
Action	What plans do you think you could make to avoid using cannabis and to avoid peer pressure? What strategy have you used or would you use to stop people from using? Are there any alternatives to cannabis use?	What do you currently do to be able to refuse consumption in these situations? Do you have friends who use? Have you suggested that they stop smoking joints? Have you succeeded? What strategy have you used so that they stop using?
Family Factors	When you go out partying or with friends, do your parents know where you are and who you are with? Are your parents aware of what you are doing?	Did your parents know where you were? Did they check your arrival time? Did they know who you were with? What other rules did you have at home? Did your parents know you were using drugs? Have these situations changed?
	What do your parents think about cannabis use? And if you consume it, how do they or do you think they would react to this situation?	What do you think your parents thought about your cannabis use? How did they react when they found out about your use? What is your parents’ attitude towards you and your achievements? Have your thoughts changed as a result of their attitude?
	Do your parents consume any substance at home (alcohol, tobacco, cannabis, etc.)? In what situations do they do it? If you use alcohol, tobacco or cannabis at home, are your parents with you? If so, in what situations or occasions? Have your parents ever offered tobacco or cannabis to you?	Do your parents use alcohol, tobacco, cannabis or other drugs? Do they use them in front of you? Have you ever used alcohol or other drugs at home? Were your parents there? Have you ever been offered alcohol or other drugs? Has your parents’ behavior changed?
	Have you ever had a conversation with your parents about substance use (alcohol, tobacco, cannabis)? What did you discuss?	What was your relationship with your parents like? How did you feel about them? Did you talk to them when you first became aware of your use problem? Were your parents the ones who made you aware that you had a problem with cannabis? How did you feel about discussing the use problem with your parents? How have these situations changed? What is your relationship with them?

Prior to conducting the Focus Groups in a high school, the center’s management was contacted and, after accepting the study proposal, a meeting was held with the teaching staff and the guidance counselor to explain the purpose of the study, so that they could inform it to the students, who were also given an information sheet. In the case of the interviews, the center that carried out the detoxification program was also contacted, to explain the purpose of the research and give them information sheets to be handed out to adolescent users and parents.

Both the interviews and Focus Groups lasted an average of about 40 min. All sessions were recorded using two independent digital recorders, upon consent of the participants, and notes were taken for transcription purposes. The saturation criterion was used to conclude data collection in both interviews and Focus Groups.

### Data analysis

All recordings were transcribed, and a deductive analysis was carried out. Through the deductive approach, the main categories were created, derived from the research question and the theoretical frame of reference. In this case, the domains of the I-Change Model were used as reference. Verbatim texts were classified with the acronym G when the discourse came from a Focus Groups plus the number identifying the group. In the case of the interviews, they were identified with the letter I, plus a number randomly assigned to each of them.

To ensure the rigor of our methodology and improve reliability [27], two researchers independently coded the transcripts. Finally, the categories, their definitions and the texts assigned to each of these categories were triangulated with the other members of the team. The coding process was carried out to the extent of data saturation and non-emergence of new themes or information.

The analysis was performed using NVIVO 11 software.

### Ethics

The ethical standards of the Declaration of Helsinki were followed [28]. In addition, the participants of both the interviews and the Focus Groups were informed that audio would be recorded and that it would only be accessible to the research team, always maintaining the anonymity of all their narrations. Participants were informed that their responses would be kept confidential to their parents and teachers. The adolescents and their parents signed an informed consent. The study was approved by the Research Ethics Committee of Andalusia, under code 0073-N-18.

## Results

Seven boys and thirteen girls (35% and 65%, respectively) whose average age was 15.5 years, participated in the Focus Groups. Of the participants, 14 lived with both of their parents and their siblings, 4 lived with just their parents, and 2 of them also lived with a grandparent.

As regards tobacco use, 25% (5) said they had tried it, and only 2 of them were regular smokers. Regarding alcohol, 55% (11) were consumers during leisure and social events, and 4 of them consumed alcohol even in front of their parents. None of the participants were regular cannabis users, although 2 reported having tried it when they were 14 and 16 years old.

As for the interviews, the participants were 7 boys and 1 girl, aged, on average, 18.1 years; 4 of them lived with both parents and their siblings, 3 with one of the parents and their siblings or grandmother or their parent’s partner, and 1 of them lived only with the mother. Regarding the level of education, 3 only completed primary school, 3 of them finished secondary school (one is currently attending the last year), 1 is in the first year of high school, and only one has completed high school. Four are currently studying and four are working.

The median age for first cannabis use was 15 years. Concerning tobacco consumption, all of them were regular smokers, and the median age of first use was 13.5 years old. In the case of alcohol, all of them consumed alcohol prior to entering the program (since no consumption is allowed in the program), with a median age of onset of 14 years. Of the participants, 1 consumed daily, 4 consumed every weekend, 1 drank one beer per week, and 2 consumed only sporadically.

### Cannabis use and characteristics of first use

Of the Focus Groups participants, Non-Regular Users (NRU), only two of them reported having tried cannabis, and none of them were regular users.

G2: *‘I have tried it, but it does not call my attention; I’m not drawn to it.’*

Some of the AYA in recovery expressed that the first time they consumed was in a festive context, with a group of friends who were consuming cannabis in the form of joints, accompanied by alcohol consumption, and that they felt curious to try it, thus reaffirming their belonging to the group.

*I5: ‘I tried it when I was 12 years old. It was because I did a “botellona” [drinking on the streets] with my friend. She was drunk and the kids were smoking joints and I told her I wanted to try it. And I did.’*

*I3: ‘When I was at school, a new kid came and he was using joints. Then many of my friends started to hang out with him. One day, when we were all drinking alcohol together, I told them to give me a puff, something that was unthinkable for me before. I used to say that I would never, ever, try drugs, but that first puff was the boost that led me to what came next. Besides, it didn’t feel bad either, in fact, I felt euphoric and happy. At that moment, you just think you’re having a good time with your friends…’*.

### Predisposing factors

This section studied predisposing factors such as gender, personal factors (personality, low self-esteem, self-confidence, self-consciousness, insecurity), or family and social factors (interfamily violence or bullying). As to gender, it is noteworthy that the majority of AYA in recovery who are enrolled in the program are men. The NRU consider that consumption is more natural among boys, who consume more quantity and more frequently, although they believe that consumption by women is becoming equal to that of men, among other things, because they are influenced by the latter.

*G3: ‘The thing is that boys do consider it to be natural, while girls may kind of hide it, and if you ask them, they say they don’t do it.’*

*G4: ‘I think boys start using it, and girls… it’s not that they follow, but they are influenced by them.’*

In the case of AYA in recovery, some think that boys consume more, but most believe that consumption is the same in terms of quantity, but the way of consuming it is what differs. Boys consume it individually, while girls do it more frequently in groups.

*I8: ‘Girls use it daily, just like boys, but in a different way. Maybe a boy has 5 € and buys it for himself and girls don’t; they get together every day, 6 or 7 of them, each with 5 € and they buy 40 € worth of joints. They smoke them and don’t leave until they have smoked it all.’*

Personal problems (self-consciousness, insecurity), or stressful family events (domestic violence) and social problems (bullying or not feeling accepted), among others, were determinant for AYA in recovery to begin consuming as a way of escaping such reality.

*I3: ‘Apart from what I have suffered, the insecurities, feeling less, not feeling accepted in a group, being bullied at school… that leads you to end up in drug situations.’*

*I7: ‘My father used to arrive home drunk very often’ ‘I consumed spontaneously, until I had a personal problem at home, which was the psychological and physical abuse… I suffered from my father. From then on, that’s when I started to use because I was… broken.’*

*I2: ‘I used to be chubby, short and they started to bully me… I also suffered it at home with my family, and that’s when we started to have problems’‘If I wasn’t in a bad place with my father, I wouldn’t have tried that because… I was afraid that he would catch me doing it and that I would have a problem, but with so many problems already, I thought What difference does it make?’*

For NRU, predisposing factors include psychological factors such as personality, low self-esteem, and low self-confidence, based on the stereotype that consumers are less socially valid. They also speak of the great accessibility to the substance.

G4: ‘But right now, people have such low self-esteem and self-confidence, that’

G1: ‘If you want, I can go right now and get you whatever you want, it’s easy, just knock on the door and that’s it.’

### Consciousness factor

This section addresses cognitive factors such as knowledge, risk perception and cues to action.

Among the cognitive factors, knowledge stands out, both of the drug itself and its effects. AYA in recovery recognize that when they started, they had little knowledge of these aspects, and this lack of knowledge is also mentioned by NRU at present.

*I4: ‘I didn’t know how to do it; I didn’t know what it was, or what substance it was, I thought it was just a cigar.’*

*G2: ‘I didn’t know cannabis was marihuana, specifically.’ “They gave talks on drugs in general, consumption and so on.’*

Once they started using, AYA in recovery do acknowledge that they learned about cannabis, both regarding the effects it produces and how to access the substance.

*I5: ‘Where it comes from, how it is made, the location of the neighborhoods where it is most sold; I don’t know, everything. I know everything.’*

Among AYA in recovery, there was little or no risk perception posed by cannabis when they first started using it.

*I4: ‘Man, you also get tired of seeing poor people, and say ‘that one is on drugs, on drugs’, but I thought ‘no, joints are like cigarettes, that one is in that situation because of the drugs, but not me because of joints.’*

This perception among AYA in recovery changes after they stop using, as they are aware of the problems it can cause in the future, associated with health, legal, violence, or social aspects (e.g., not finishing school). NRU have a similar discourse, focusing the consequences on the lack of impulse control, less capacity for work and difficulties in relationships.

*I1: ‘I don’t see anything positive about drugs right now. Because they give you three hours of freedom, then the weight falls on you and it is twice as much.’ ‘To lose my relationship with my family, again, to get bad grades, again, to hang out with people I shouldn’t, again.’*

*I3: ‘It affects you at a psychological level, and your entire body. You can feel it, it’s hard to react, and it’s hard to have conversations’*.

*G4: ‘The relationship with your parents; family life would not be the same.’*.

Among the NRU, the risk perception is mainly related to the amount and time of consumption, justifying that if they abuse it they can become addicted, but not by trying it or consuming it occasionally.

*G1: ‘If you smoke one joint a month, maybe nothing happens to you; it can even be good, but what happens is that if you smoke one, you will not smoke just one, you will end up smoking more and that will cause lung cancer, and even more things; you will become addicted.’*

Regarding which they consider to be more dangerous (tobacco or cannabis), NRU say that they believe cannabis is more dangerous because it is a drug and causes more damage in the short term, and that tobacco consumption is more natural in social terms.

*G4: ‘Cannabis can be more dangerous, like more… a little more aggressive.’*

In contrast, we found that one of the AYA in recovery today considers tobacco use to be more dangerous than cannabis use because the former can kill you, while the latter only affects you at a psychological level.

*I7: ‘I honestly believe tobacco is more dangerous, because it kills you physically. Cannabis, I think, kills your neurons; you get stupid, but it doesn’t kill you like tobacco; you can get lung cancer and pass away.’*

According to AYA in recovery, entering the detox program has helped make the rest of the group of friends aware of the consequences of consumption, and they assure that this fear has influenced peers to quit, as a cue to action.

*I3 ‘As I came here, most of my group have stopped using because they got scared after seeing me like this.’*

### Motivational factors

Within motivational factors, attitude (advantages and disadvantages of consumption), social influence (social modeling, social norms and social pressure) and self-efficacy have been explored.

#### Attitude

Among the advantages, both AYA in recovery and NRU agree that cannabis produces a feeling of freedom and escape while under its effects. According to this, it makes relationships easier and is fun; in short, it makes them feel good.

*I1: ‘Freedom to do what you want without thinking about the consequences. To interact, to talk to anyone, to enjoy yourself, to feel good…’*.

*G4: ‘I think they use it as an avoidance method, to… if you have problems or whatever, you use it, and you don’t care about anything.’*

NRU and AYA in recovery also agree on the disadvantages: lack of money and the deterioration of their social relationships when consumption is regular, both due to the lack of interest of the consumer and the rejection of the environment, or deterioration of interfamily relationships.

*I4: ‘If you wanted to go to the movies or buy a hamburger for one euro, or if you needed money for whatever, you had no money at all, nothing.’*

*I3 ‘You don’t care about anyone; you have no empathy. You are insecure, unhappy.’*

*G4: ‘You may steal from your parents or have problems with them.’*

AYA in recovery say that when consuming, they are “forced to change friends”, since their lifestyles become different.

*I3: ‘I have changed my friends, because at the beginning I used to hang out with kids from my school, but then I stopped hanging out with them because they didn’t have my same lifestyle.’*

However, this group admits that, at the beginning of consumption, they were not able to see these disadvantages.

*I3: ‘Back at that time, I mostly saw the advantages of drugs.’*

#### Social Influence

Regarding social modeling, among AYA in recovery we found that some of them state that some family members and friends are cannabis users, or have recently quit. We also found the case of a family member who used cocaine.

*I5: ‘I have friends from before, some of whom have stopped smoking joints and others are still using.’*

*I3: ‘One of them who is still my best friend (is still using cannabis). He is in a very complicated situation and I am trying to help him.” ‘My uncle on my father’s side used cocaine.’*

*I2: ‘An aunt, but I am not supposed to know. Nobody knows I know.’*

Among NRU, we also found two participants with a family member currently undergoing rehabilitation for use of this substance.

*G2: ‘My cousin at the moment is in the men’s project, but he is still using.’*

If we refer to social norms, according to AYA in recovery, cannabis use was mainly approved by the peer group of consumers.

*I8: ‘My friends, but out of inexperience, but a bit more, just my friends.’*

*I2: ‘My girlfriend (at that time), and all my friends.’ ‘They didn’t say anything to me since I didn’t hang out with anyone who was not a user.’*

NRU say that consumers look for their leisure group among those who have their same consumption habits.

*G2: ‘Because they no longer hang out with the people they used to hang out with, and they start getting together more with smokers.’*

As regards social pressure, both groups (AYA in recovery and NRU) agree that they have not felt pressured to consume cannabis, but that it may influence consumption if friends are involved in it, as they believe that, this way, they will be more accepted by the group.

*I4: ‘He didn’t force me or pressure me or anything. Nobody pressures you, normally, because drugs are very expensive.’*

*I1: ‘Well, an environment in which your friends smoke and you are the one who doesn’t. At that moment, if I offer you and you say no, maybe you think ‘I might look badly, maybe he’ll stop hanging out with me because I don’t do what he does.‘’*.

*G4: ‘I was offered, but not pressured.’*.

In contrast to this, there is a AYA in recovery who says he did not feel pressured the first time, but later he did, and it could have influenced the consumption chronification.

*I2: ‘Not the first time, but then, I did.’ ‘They found out that I had already smoked once, and they started saying ‘come on, you’ve already smoked once’. They did that every day and I was hanging out with those people, so I started smoking, and smoking, just because I wanted to.’*

Some AYA in recovery also do not feel pressured to consume after quitting; they believe that they are respected, although they emphasize that if someone becomes really insistent, they would end the relationship with that person.

*I1: ‘I have explained my situation to them, and some have understood me and others have not, and continued to insist. And when they kept doing so, I told them: ‘look, let’s quit it now’ and the relationship ended.’*

#### Self-efficacy

Both groups (AYA in recovery and NRU) agree that, when they get together to have fun, at a party or with a group of friends, it is hardest for them to refuse drugs, especially when they are offered by several people.

*I6: ‘For example, I don’t go to places where there are people smoking.’ ‘Well, parties, for example… parties are very dangerous. That’s where I’ve consumed the most. Right now I cannot go to parties.’*

*G4: ‘At some party, with a friend who smokes joints.’*

NRU also highlight other situations in which it is difficult for them not to consume, such as peer pressure or because they want to be accepted by another person. Some AYA in recovery mention stress or anger as factors that make it difficult to refuse consumption.

*G4: ‘When you like a boy and you want to become part of the group of friends, and he offers you and says ‘come on, don’t be…’*.

*I3: ‘When I was angry I used to consume all the time, it is hard to say no when you feel like that.’*

### Action

In the action section, plans to avoid consumption and skills/barriers to implement and keep those plans on track have been explored. The strategies used by AYA in recovery to avoid consumption are social support, for example, from a partner, avoidance, such as leaving the place where they are consuming and looking for entertainment alternatives:

*I4: ‘Being with my girlfriend, in the sense that if I feel like that, longing for that. I just walk away and if not, I tell my girlfriend, and we look for a solution; we just walk away.’*

*I1 ‘For example, now I go to the gym, ride horses, go for bike rides, and play soccer. I don’t know, it’s just entertainment.’*

NRU also mention avoidance and seeking alternatives as strategies, just like AYA in recovery, and add self-control and conviction in decisions, as well as showing an assertive behavior.

*G1: ‘A person who does not want to, no matter how much they insist, is not going to do it.’*

*G4: ‘Going to other places, for example.’*

AYA in recovery consider that there must be an internal motivation to quit using drugs; they must want to stop.

*I8: ‘It is something that, as I said, you have to see for yourself.’*

NRU express that, once consumption is established, they believe it is difficult for someone to stop using just because they are lectured about the subject.

G4: *‘I believe that it cannot be changed, just as they cannot convince me that it is good, I don’t think they can be convinced either.’*

One aspect that came up as an element to successfully continue staying drug-free, in the case of AYA in recovery, is the thought that if they relapse they would hurt people who are close to them, such as family or a partner, and this makes them stay away from consumption when they feel like doing it.

*I6 ‘I think of my parents, first. They are dealing with a lot of problems.’*

### Family factors

#### Parental supervision

Parental supervision was a topic addressed in the interviews. Two of the AYA in recovery report that before their parents found out they were using, they were very permissive in terms of schedules and rules.

*I6: ‘It was my free will; they would pick me up at whatever time I wanted: I did everything I wanted.’*

The rest of the AYA in recovery group recognizes that the rules were mainly focused on academic performance. This was of outmost importance, and what they were held accountable for.

*I1: ‘If you study and you pass, there are no rules; if you fail, then you are grounded.’*

Something striking in the discourse of the NRU is that they consider that, if parents are too permissive or too controlling, it can lead to a higher risk of cannabis use.

G3: *‘I know people who are really, really controlled, and when they let them go out a little bit, they do everything that their mother won’t let them do.’*

The AYA in recovery in the rehabilitation program are aware that rules are stricter, and consider that they are subject to more control.

G3: *I4 ‘Now that I am in the project, I am more controlled.’*

In general, AYA in recovery recognize the control of their parents, especially through the telephone, since it is one of the most used tools during the detox treatment. Most of them feel that their parents are very aware of what they are doing.

*G2: ‘Well, I always convince her to let me come home later, but she always checks up on me; she worries a lot.’*

#### Parents’ support

Regarding the perception of NRU of the parents’ support for their consumer children to stop using, they say that they are only aware of it when they are in extreme situations and that, in addition, they lack knowledge of the available resources.

G3: *‘I think it is because they think it is pointless. Because if you look for help, no rehabilitation center will tell you not go in… but because they think it is easier for the child to continue doing whatever he or she wants.’*

For the AYA in recovery, their parents were shocked when they found out about their drug use, and they say that they supported them to stop using. It is true that some of them express, in agreement with what NRU say, that their parents suspected their consumption, but they were not aware of the seriousness of the situation.

*I3: ‘They imagined that I was fooling around with drugs, but not to the extent I had reached.’ ‘Their reaction was shocking; they were really surprised.’*

When asked how their parents would react if they were using, the NRU said that they would be very angry.

I*G3: ‘My mother would kill me when I get home.’*

We can thus affirm that, for both groups, the opinion or influence of parents/guardians is key, both for cannabis consumption and for not doing so.

I*2: ‘Well, I am grateful (to my parents) that they insisted and insisted. Other parents say ‘if he smokes, then let him smoke’ and I would still be in the street smoking and doing whatever I want.’*

When asked what their parents think of them now, AYA in recovery consider they have a positive opinion, and the new situation makes them happy, proud or confident.

*I5: ‘Very proud of me; they see that I have matured a lot, that I am not the same girl as before.’*

*I1: ‘I see she is happy; she trusts me; she’s cheerful.’*

Communication among the family of the AYA in recovery before entering the program was scarce; they hardly spoke with their parents.

*I1: ‘We had no communication, affection before, that is, there was no relationship at all. There was an apathetic relationship on both sides, on my mother’s side and mine.’*

However, nowadays this situation has changed, the relationship improved, and there is more communication.

*I3: ‘I have a polite relationship with my parents, I talk to them. I go out and come home with them, and we go out for drinks. Besides, I am the one who takes the initiative.’*

#### Alcohol and tobacco in the family

They were also asked about the consumption of alcohol and tobacco in the family. Both groups agree that, of both parents, fathers consume these substances more frequently.

*I7: ‘My mother used to drink beer, but only during meals, but I see that as normal; every time she eats, she has a beer… typical of Andalusia.’*

*I8: ‘Yes, when my father goes out partying, he has a beer and something more, but he never drinks at home on a daily basis. And neither does my mother.’*

Both groups state that they have consumed alcohol in front of their parents, but only at celebrations, and that their parents allow them to drink in front of them to check that they are okay after doing so.

*I3: ‘Yes, a few times. At communions and weddings, for example.’*

*G4: ‘In front of them; my father says ‘I’d rather give you a drink and see how you are.’ ‘My mother says ‘come on, have a little drink…’*.

## Discussion

The aim of this study was to get to know the experiences and opinions of adolescent cannabis non-users and regular users about its use and the factors that determine it, using the I-Change explanatory model as a basis, focusing on predisposing, awareness and motivational factors. In addition, the influence of parents and the first consumption experience were studied.

### Predisposing factors

Regarding predisposing factors, age, gender, as well as personal problems (personality, low self-esteem and self-confidence, self-consciousness or insecurity, social issues (bullying or lack of acceptance) and family problems (even observing cases of interfamily violence) stand out, which should be considered when developing prevention programs aimed at the most vulnerable population [22–24]. A recent study shows the relationship between drug use to cope with problems and a higher risk of problem consumption [29], which is consistent with the causes of the onset of consumption expressed by AYA in recovery and NRU. As for gender, the predominant opinion is that boys use it more frequently, but the AYA in recovery express that the difference lies in the use pattern rather than the quantity. The lower levels of cannabis use by females could be due to the higher risk perception of cannabis by females than by males [27], since adolescents who have a higher risk perception are less likely to start using cannabis. Despite the above, it should be noted that cannabis use is increasing among girls [4, 30]. This finding, in terms of the gender perspective, is consistent with previous research and should be used for program design purposes [3, 4].

### Consciousness factor

In terms of awareness factors, there is a lack of knowledge about the substance among NRU, as was the case of AYA in recovery before they started using it. They also agree on the consequences of cannabis use: health, social, educational and even legal problems. AYA in recovery did not have a risk perception when they started using the substance. The lack of knowledge on cannabis by NRU and AYA in recovery before use is consistent with what has been reported in other published studies in which participants had little information, or even incorrect information, about cannabis [31]. The consequences of consumption perceived by both AYA in recovery and NRU mostly focus on the psychological and social aspects, ignoring the physical consequences, such as arrhythmias or bronchopulmonary diseases [32]. The low risk perception by AYA in recovery participants is in line with that found in previous studies [33, 34], also increasing its use probability [35]. Therefore, it is worthwhile to continue intervening on these factors in prevention programs.

When they quit smoking cannabis, the AYA in recovery perception of risk changed. Some authors linked negative expectations about cannabis with a greater predisposition to stop using [36] and a lower risk of relapse [37, 38]. In the study conducted by Herruzo et al. [39], and the ESTUDES report [4], participants (young people aged 18–29) considered tobacco to be more harmful than cannabis, a belief that is similar to that of one of our interviewees in the AYA in recovery group, but contrasts with what NRU state, who consider cannabis to be a more dangerous substance, perhaps because no member of this group is a regular user of cannabis or tobacco. In our sample, all AYA in recovery are tobacco users, which coincides with data from the ESTUDES study where 1.4% of non-cannabis users smoked daily, increasing this figure to 38.2% in the case of cannabis users [4]. This co-use of tobacco and cannabis has been seen in different research studies, so it is important to monitor this co-use and develop prevention interventions aimed at it [40–42].

### Motivational factors and action

Regarding motivational factors, we found that the main advantages of cannabis use for our participants were feeling freedom and avoiding stressful events. No studies were found that specifically discussed the feeling of freedom, but the avoidance of stressful events is often related to use, as they tend to be more common in regular cannabis users who reported having experienced more negative life events than non-users [43]. The most frequently mentioned disadvantages were lack of money and deterioration of social relationships. Therefore, it is essential to take these cognitions into account in order to address them in consumption prevention programs, emphasizing the disadvantages and providing alternatives to the advantages observed.

The social influence exerted by the peer group stands out, which has been widely referred to by previous studies [44, 45] as a strong predictor of the onset of cannabis use, since in adolescence, belonging to a group plays a crucial role [46].

In the case of self-efficacy, both NRU and AYA in recovery expressed that the time when it is easiest for them to consume cannabis is at parties, together with the peer group. This is reflected in the ESTUDES report [4], where almost 50% of those who had used cannabis had done so during a *botellón* [drinking in the streets]. This should be taken into account when developing health education advice that includes specific actions to deal with these situations. In fact, both groups agree that the best way not to consume is to avoid going to places where there are consumers, to look for leisure alternatives, and to rely on people who are close to them. In a study carried out in Spain with university students, social support and, specifically, perceived acceptance were considered to be protective factors against challenging situations such as relapse or start of consumption [47].

### Family factors

Focusing on family factors, when AYA in recovery started using, they had poor relationships, few rules and lack of parental supervision. This was not the case for NRU, but they expressed the importance of parents in the prevention of consumption or quitting. Support and favorable relationships with parents are linked to a lower risk of drug and alcohol use, while lack of supervision or excessive supervision has been considered a risk factor for substance use [48, 49].

With respect to communication with parents, AYA in recovery stated that before and during consumption, communication was poor, although currently it is much better. In the study by Herruzo et al. [39], the perception of risk was higher in those adolescents who had received information about cannabis from their parents.

In both groups, one of the parents consumed alcohol and/or tobacco, mostly fathers. Family consumption of substances such as alcohol and/or tobacco has been shown to be a factor influencing the consumption of these substances by youngsters [50]. Having a parent who smokes influences the smoking of children [51], and something similar occurs with alcohol, since the consumption pattern of the parents has an impact on their children’s potential consumption [52, 53]. Consumption of other substances such as alcohol and tobacco at an early age has been associated with a greater likelihood of consuming substances such as cannabis [54].

## Strengths and limitations

Among the strengths of the study, the discourses of both AYA in recovery and NRU have been collected and compared, allowing to obtain a broader view of the problems studied. The data obtained in this research are consistent with the I-Change model. Also, this is the first research of its kind in Andalusia, based on a theoretical model of behavior, which can be a key element for the development of prevention programs in this population. In future interventions, it would be advisable to use a behavior change prediction model such as the I-Change model as a theoretical framework, which has proven to be useful. Furthermore, when developing and launching prevention health messages, it would be interesting to take into account and use as examples those referred to by adolescents themselves in studies such as ours. In addition, the use of a qualitative methodology has allowed us to deepen the explanation of the phenomenon from the perspective of the social players themselves.

Regarding limitations, in the Focus Groups, group pressure and being on the same class could inhibit some participants. This was avoided by ensuring the confidentiality of discussions and by encouraging everyone to participate, reminding them that there are no wrong answers. The fact that there were not many opinions of girls in the AYA in recovery group can be considered a limitation, since their arguments could have contributed another perspective to the interviews. This study has not analyzed informational factors that should be addressed in another study. The research has focused on personal, group and family aspects related to consumption. We could consider the age difference between the focus group participants and individual interviews as another limitation. Most of the individual interviewees are older than the adolescents in the focus groups, but they all started using at the age of NRU. So the perception of AYA in recovery about cannabis when they were using may be similar to that of NRU.

Our study reflects perceptions in a specific context and environment. More research is needed to elicit views in different environments.

## Conclusions

As regards predisposing factors, it was found that personal problems (personality, self-esteem, self-confidence and insecurity), social problems (such as bullying) or family problems (domestic violence) can lead to cannabis use. In addition, there is a consumption pattern associated with the male sex that tends to change. As for the cognitive factors, we found a lack of knowledge and low risk perception about consumption, whereas motivational factors showed the perception of advantages, such as the feeling of freedom, the influence of the peer group or the lack of emotional or situational self-efficacy in the face of events that entices them to consume. In addition, action plans to avoid substance use have been identified, as well as the role of the family. Knowledge of these factors is of vital importance as a prior step to the development of efficient intervention measures adjusted to the needs identified and the characteristics of the population.

## Data Availability

The datasets used and/or analysed during the current study are available from the corresponding author on reasonable request.

## Abbreviations

NRU: Non-regular cannabis users
AYA: Adolescents and young adults
